# Inequalities in socio-economic characteristics and health and wellbeing of men with and without disabilities: a cross-sectional analysis of the baseline wave of the Australian Longitudinal Study on Male Health

**DOI:** 10.1186/s12889-016-3700-y

**Published:** 2016-10-31

**Authors:** Anne M. Kavanagh, Zoe Aitken, Eric Emerson, Sash Sahabandu, Allison Milner, Rebecca Bentley, Anthony D. LaMontagne, Jane Pirkis, David Studdert

**Affiliations:** 1 Centre for Health Equity, Melbourne School of Population and Global Health, The University of Melbourne, Melbourne, 3010 Australia; 2 Centre for Disability Research and Policy, Faculty of Health Sciences, University of Sydney, Lidcombe, 2141 Australia; 3 Centre for Disability Research, Faculty of Health and Medicine, Lancaster University, Lancaster, LA1 4YG UK; 4 Centre for Epidemiology and Biostatistics, Melbourne School of Population and Global Health, The University of Melbourne, Melbourne, 3010 Australia; 5 School of Health & Social Development, Deakin University, Burwood, 3125 Australia; 6 Centre for Mental Health, Melbourne School of Population and Global Health, The University of Melbourne, Melbourne, 3010 Australia; 7 Centre for Health Policy/PCOR, Stanford University School of Medicine, Stanford, 94305 CA USA; 8 Stanford Law School, Stanford, 94305 CA USA

## Abstract

**Background:**

Internationally, men with disabilities have higher rates of social and economic disadvantage and poorer health and wellbeing than men without disabilities. No single study has provided comprehensive, population-level information about the magnitude of such differences among adult men using a well-validated instrument to measure disability.

**Methods:**

We analysed baseline data from Ten to Men – an Australian longitudinal study of male health. Ten to Men used a stratified multi-stage cluster random sample design to recruit a national sample of males aged 10 to 55 years residing in private dwellings. Data were collected between October 2013 and July 2014 from 15,988 males. This analysis was restricted to 18–55 year old participants with data available on age and disability (*n* = 13,569). We compared the demographic, socio-economic characteristics and health and wellbeing of men with and without disabilities using chi squared tests for proportions and t tests for continuous variables. Linear regression adjusted for age was used to assess the association between disability status and health and wellbeing, which were measured using the SF-12 mental and physical health component scores and the Personal Wellbeing Index.

**Results:**

Men with disabilities were older and more likely to be born in Australia, speak English at home, be Aboriginal and Torres Strait Islander and were less likely to be married or de facto, or to live in urban areas. They were less likely to have completed secondary school, be employed and live in affordable housing, and were more likely to live on low incomes, in more socio-economically disadvantaged areas, and in rental accommodation and to experience shortages of money. Among employed men, those with disabilities were less likely to be in high skilled jobs, worked less hours on average, and were more likely to report that they would prefer to work more. Men with disabilities had lower levels of social support and community participation and poorer mental and physical health and overall wellbeing.

**Conclusion:**

Adult men with disabilities experience marked social and economic disadvantage and poorer health and wellbeing. Improving the health and wellbeing of disabled men should be a priority for public health researchers and policy-makers.

**Electronic supplementary material:**

The online version of this article (doi:10.1186/s12889-016-3700-y) contains supplementary material, which is available to authorized users.

## Background

Australians with disabilities have significantly worse life outcomes than their peers without disabilities. They have lower levels of social and economic wellbeing and poorer health on a variety of health status measures, including ones unrelated to their impairment [1, 2]. For example, people with disabilities are more likely to be overweight or obese, smoke, be physically inactive and have poor diets; they also have poorer self-rated health, life satisfaction, and mental health [3–12]. They have higher rates of chronic conditions such as diabetes and heart disease and are more likely to use clinical services but less likely to use preventative health care [8, 13–17].

The inequalities extend beyond direct measures of health and wellbeing. Compared to Australians without disabilities, Australians with disabilities have lower levels of employment, education, income, community participation and social support, and higher levels of housing stress, poverty and inter-personal violence [10, 12, 18–20]. Inequalities between people with and without disabilities in Australia are starker than in other Organisation for Economic Cooperation and Development (OECD) countries. Relative to Australians without disabilities, the income of Australians with disabilities is the lowest of all OECD countries and they have one of the lowest levels of labour force participation [21]. Analyses of national data from the Australian Survey of Disability, Ageing and Carers involving over 30,000 adults revealed that men with disabilities were more likely to experience concentrated disadvantage (i.e. multiple indicators of disadvantage such as low income, housing stress and unemployment) than men without disabilities (22 % vs 5 %) [22]. The disadvantaged circumstances of men with disabilities are likely to have flow on effects to health and wellbeing. Emerging evidence suggests that socio-economic disadvantage makes a substantial contribution to disability-related inequalities in health [23].

There is an absence of comprehensive analyses of the social, economic and health and wellbeing outcomes between Australian men with and without disabilities that is in part due to the limitations of current studies. Health-related studies do not collect adequate data on disability and rarely include measures of wellbeing. The main source of data on disability in Australia is the Australian Bureau of Statistics (ABS) Survey of Disability, Ageing and Carers, but it has limited socio-economic data on people without disabilities and does not collect information on health [20].

This paper used data collected from adult men who participated in the first wave of the Australian Longitudinal Study of Male Health (Ten to Men) – a large nationally representative study of Australian boys and men which included the Washington Group short set of questions on disability, an internationally validated instrument to measure disability [24]. We capitalised on the unique combination of disability, social determinants and health and wellbeing data collected in the baseline wave to provide a comprehensive comparison of a large sample of adult men both with and without disabilities across a range of social, economic, and health and wellbeing indicators.

## Methods

### Data source

Ten to Men is a national longitudinal study of boys and men aged 10 to 55 years. In waves spaced approximately 2–3 years apart, we will collect data on a range of life domains, including demographic and socio-economic characteristics, physical and mental health and wellbeing, health behaviours, and use and knowledge of health services. The baseline survey was conducted between October 2013 and July 2014. The total sample consisted of 15,988 males, who were sampled using a multi-stage, clustered, random sample design. All eligible males residing in private households were approached, with separate cluster samples drawn from regional strata in order to oversample males from regional areas. Data were collected from males 15 years and older using a self-completion questionnaire and by interview and parent self-completion questionnaire for boys aged 10 to 14 years. The response fraction was 35 %. Response rates for individual questions in the survey varied between 82 and 100 %. More details on the study can be found elsewhere [25].

The sample for this analysis consisted of 13,569 of the 13,884 participants aged 18 to 55 years and excluded those who did not report their age (0.9 %) or did not respond to the disability questions (1.4 %).

#### Disability variable

The Ten to Men survey included the Washington Group short set of questions on disability, a widely used instrument for measuring disability which has been validated in many countries [24]. It consists of six questions relating to core functional domains (seeing, hearing, walking, cognition, self-care and communication) with answers reported on a severity scale (no difficulty, some difficulty, a lot of difficulty and cannot do at all). We classified participants as having a disability if they reported that they had a lot of difficulty or cannot do at all on one or more of the core domains [24].

#### Other variables

The survey included questions on a range of demographic and socio-economic variables including age, race/ethnicity, relationship status, education, employment, housing, income and financial security. Age was classified into the following groups: 18 to 24, 25 to 34, 35 to 44, and 45 to 55 years. Variables measuring race/ethnicity included country of birth (Australia, other); language spoken at home (English, other); and Aboriginal and Torres Strait Islander (ATSI, not ATSI). Relationship status was coded as married or in a de facto relationship, separated or divorced or widowed, and single. Area of residence was defined as metropolitan, inner regional, and outer regional. Socio-economic variables included education (did or did not complete secondary school); household income (≥$150,000, $100,000 to $149,999, $50,000 to $99,999, $30,000 to $49,999, $1 to $29,999, nil or negative income), and labour force status (employed, unemployed and looking for work, and not in the labour force). If employed, occupation was classified according to skill level (high, medium and low skill); employment arrangements (permanent, fixed-term or casual, self-employed); number of hours worked; preference for hours worked in relation to current hours worked (about the right amount, prefer fewer hours, prefer more hours); access to paid leave (e.g. sick or annual leave) (yes, no); experienced shortage of money in the last 12 months (yes, no - based on whether they answered yes to one or more of six questions regarding financial security such as could not pay bills on time); housing tenure (outright owner, mortgagor, living in rental accommodation, other); and housing affordability (affordable, unaffordable - defined as paying more than 30 % of household income on accommodation).

Area-level socio-economic disadvantage was categorised using population quintiles of the Index of Relative Socio-economic Disadvantage (IRSD), measured at Statistical Area Level 1 (SA1), the smallest geographic unit in the Australian Standard Geographic Classification. The IRSD is a composite index that summarises a range of information about the economic and social conditions of the people living within specific geographic areas [26]. A low IRSD score indicates relatively greater disadvantage.

Social support was measured using eight questions relating to emotional and informational support developed for the Medical Outcomes Study [27]. The questions elicit the availability of different kinds of support, which is graded on a five point scale ranging from “none of the time” to “all of the time”. Responses to these questions were summed and described as a continuous variable. Social participation was measured using three variables: current active membership in a club or association (yes, no), participation in ongoing community service activities (yes, no), and frequency of attendance at events that bring people together, such as fetes, festivals and community events (very often/often/sometimes, rarely/never). Participants were also asked about *any* experience of discrimination over the past two years, which was coded as no (never) or yes (rarely, occasionally, fairly often, very often).

Several of the many measures of general health and wellbeing collected in Ten to Men were used in this analysis. The Physical Component Summary (PCS) and the Mental Component Summary (MCS) scores are summary measures of physical health and mental health, respectively, derived from the Short Form 12 (SF-12) health survey [28]. The summary scores are validated, psychometrically based measures that are standardised to a mean of 50 and standard deviation of 10, and a range from 0 (worst health) to 100 (best health). The Personal Wellbeing Index – Adult (PWI-A) is a multi-item scale which measures subjective wellbeing [29]. The PWI-A is based on responses to seven questions that measure satisfaction within specific life domains: standard of living, health, life achievements, personal relationships, safety, feeling part of a community, and future security. Responses are recorded on an 11-point Likert scale ranging from 0 (completely dissatisfied) to 10 (completely satisfied). We analysed the seven domains individually and in aggregate via the PWI-A, which is created by summing the seven items to yield an overall score of subjective wellbeing.

#### Statistical analysis

First, we calculated the population-weighted prevalence of disability among Australian men aged 18 to 55 years, by age group. We compared the patterns of demographic, social and economic variables between men with and without disabilities using proportions and means, and tested for differences using Chi squared and *t* tests respectively. To ensure that any differences in health and wellbeing between the groups were not confounded by systematic differences in the distribution of age, we also used population weighted linear regression analysis that adjusted for age.

The sample weights used in the weighted estimates (prevalence and age-adjusted health and wellbeing comparison) were calculated based on the inverse of the probability of selection at the level of the individual participant [30].

The number and proportion of missing observations for each variable are described in Additional file 1: Table S1.

## Results

### Demographic characteristics

In total, 957 men in the sample were classified as having a disability, a population weighted prevalence of 6.8 % (95 % CI 6.8 %, 6.8 %). The population-weighted prevalence of disability was 6.4 % (95 % CI 6.3 %, 6.4 %) among men aged 18–24, 5.9 % (95 % CI 5.9 %, 6.0 %) among 25–34 year olds, 6.0 % (95 % CI 6.0 %, 6.1 %) among 35–44 year olds and 8.4 % (95 % CI 8.4 %, 8.5 %) among 45–55 year olds.

Compared to men without disabilities, men with disabilities were older and were more likely to be born in Australia, speak English at home, and be ATSI (Table 1). On the other hand, men with disabilities were less likely to be residing in metropolitan areas or living in a married or de facto relationship.

**Table 1:** Demographic characteristics of men with and without disabilities

	Disability		No disability		*P* value
	*N*	%	*n*	%	
Age					
18–24 years	126	13.4	1844	14.6	*p* < 0.001
25–34 years	190	20.2	2866	22.7	
35–44 years	242	25.7	3833	30.4	
45–55 years	384	40.8	4084	32.3	
Mean, SD	39.5	11.0	38.0	10.6	*p* < 0.001
Country of birth					
Australia	775	82.3	9597	76.0	*p* < 0.001
Other	167	17.7	3030	24.0	
Language spoken at home					
English	870	94.2	11,405	91.3	*p* = 0.003
Other	54	5.8	1081	8.7	
Indigenous status					
Not ATSI	888	95.1	12,308	97.9	*p* < 0.001
ATSI	46	4.9	267	2.1	
Relationship status					
Married/de facto	518	55.7	8501	67.8	*p* < 0.001
Separated/widowed/divorced	118	12.7	773	6.2	
Single	294	31.6	3261	26.0	
Area of residence					
City	469	49.8	7427	58.9	*p* < 0.001
Inner regional	262	27.8	2817	22.3	
Outer regional	210	22.3	2374	18.8	

### Socio-economic characteristics

Men with disabilities were more disadvantaged than non-disabled men on all but two of the 12 socio-economic variables analysed (Table 2). They were less likely to have completed secondary school (39 % vs 62 %) and live in affordable housing (55 % vs 63 %) and were more likely to live on low incomes, in more socio-economically disadvantaged areas (28 % vs 18 %), live in rental accommodation (41 % vs 29 %) and to experience shortages of money (55 % vs 30 %).

**Table 2:** Socioeconomic characteristics of men with and without disabilities

	Disability		No disability		*P* value
	*n*	%	*n*	%	
Education					
Completed secondary	357	39.4	7598	61.9	*p* < 0.001
Did not complete secondary	549	60.6	4678	38.1	
Household income (annual)					
≥ $150,000	92	11.8	2417	22.3	*p* < 0.001
$100,000–$149,999	124	15.9	2901	26.8	
$50,000–$99,999	268	34.4	3773	34.8	
$30,000–$49,999	142	18.2	1108	10.2	
$1–$29,999	144	18.5	561	5.2	
Nil/negative	9	1.2	70	0.7	
Labour force status					
Employed	578	63.1	10,808	87.2	*p* < 0.001
Unemployed	127	13.9	984	7.9	
Not in the labour force	211	23.0	597	4.8	
Skill level^a^					
High	142	26.4	4022	39.1	*p* < 0.001
Medium	209	38.8	3692	35.9	
Low	187	34.8	2581	25.1	
Employment arrangements^a^					
Permanent	378	67.1	7447	70.0	*p* = 0.113
Fixed term/casual	99	17.6	1540	14.5	
Self employed	86	15.3	1646	15.5	
Number of hours worked^a^					
Mean, SD	39.3	19.3	41.7	15.9	*p* < 0.001
Hours of work preferred^a^					
About right amount	243	43.9	5606	52.7	*p* < 0.001
Prefer fewer hours	179	32.3	3357	31.6	
Prefer more hours	132	23.8	1673	15.7	
Access to paid leave^a^					
Yes	312	65.0	6217	68.5	*p* = 0.111
No	168	35.0	2862	31.5	
Shortage of money					
No	410	45.1	8612	70.2	*p* < 0.001
Yes	500	55.0	3663	29.8	
Housing tenure					
Outright owner	134	14.8	1881	15.2	*p* < 0.001
Mortgagor	341	37.7	6302	51.1	
Rented accommodation	371	41.0	3530	28.6	
Other	59	6.5	626	5.1	
Housing affordability					
Affordable	425	55.2	6800	63.2	*p* < 0.001
Unaffordable	345	44.8	3960	36.8	
SEIFA					
Q5 (less disadvantaged)	102	10.8	2406	19.1	*p* < 0.001
Q4	152	16.1	2759	21.9	
Q3	198	21.0	2957	23.4	
Q2	226	24.0	2279	18.1	
Q1 (more disadvantaged)	264	28.0	2221	17.6	

^a^Among employed men

Men with disabilities were much less likely to be employed than their non-disabled counterparts (63 % vs 87 %). Among those who were employed, men with disabilities were less likely to be in high skilled jobs (26 % vs 39 %), worked fewer hours per week (on average 39 vs 42 h) and were more likely to report that they would prefer to work more (24 % vs 16 %). However, there were no statistically significant differences between workers with and without disabilities with respect to employment arrangements and access to paid leave.

### Social support, participation and discrimination

Men with disabilities had lower levels of social support (mean score of 60 vs 71), were less likely to be a member of club or association (29 % vs 39 %), to have participated in community service (19 % vs 25 %), and to attend community events (26 % vs 37 %), whereas they were substantially more likely to have experienced discrimination (61 % vs 46 %) (Table 3).

**Table 3:** Social support, participation, and discrimination of men with and without disabilities

	Disability		No disability		*P* value
	*n*	%	*n*	%	
Social support					
Mean (SD)	60.1	30.0	70.5	26.1	*p* < 0.001
Group membership					
Yes	268	29.4	4758	38.9	*p* < 0.001
No	643	70.6	7472	61.1	
Community service					
Yes	171	18.8	3081	25.2	*p* < 0.001
No	739	81.2	9155	74.8	
Community events					
Sometimes/very often	234	25.7	4573	37.3	*p* < 0.001
Never/rarely	677	74.3	7698	62.7	
Discrimination					
No	347	39.0	6612	54.4	*p* < 0.001
Yes	544	61.1	5540	45.6	

### Health and wellbeing

For every one of the health and wellbeing measures examined, mean scores were lower among men with disabilities than they were among men without them (PCS: 45.5 vs 54.5; MCS: 41.5 vs 50.5; PWI-A: 54.4 vs 71.5) (Table 4). For the variables describing satisfaction with each of the life domains, there was a 1.3 to 2.3 point difference between people with and without disabilities.

**Table 4:** Health and wellbeing of men with and without disabilities

	Disability		No disability		*P* value
	Mean	SD	Mean	SD	
SF-12 Physical Component Score	45.4	11.8	54.5	6.6	*p* < 0.001
SF-12 Mental Component Score	41.5	11.9	50.5	8.7	*p* < 0.001
Personal Wellbeing Index	54.4	21.8	71.5	16.2	*p* < 0.001
Satisfaction with life domains					
Standard of living	5.9	2.6	7.4	2.0	*p* < 0.001
Health	4.5	2.6	6.8	2.0	*p* < 0.001
Life achievements	4.8	2.9	6.8	2.2	*p* < 0.001
Personal relationships	6.2	3.1	7.5	2.4	*p* < 0.001
Safety	7.0	2.8	8.3	1.8	*p* < 0.001
Feeling part of a community	5.1	2.9	6.8	2.3	*p* < 0.001
Future security	4.8	3.1	6.8	2.4	*p* < 0.001

Men with disabilities scored 16.8 points lower (95 % CI −18.7, −14.9) on the PWI-A after adjusting for age in the regression analyses, the PCS score was on average 9.4 points lower (95 % CI −10.5, −8.4) and the MCS score was 8.8 points lower (95 % CI −9.8, −7.7). Overall adjusting for age had trivial effects on the size of these differences (see Additional file 2: Table S2 for coefficients).

## Discussion

This analysis of data gathered in a large national study of adult Australian men showed that men with disabilities had higher levels of social and economic disadvantage and poorer health and wellbeing than their non-disabled peers. They were disadvantaged on most socio-economic indicators across all domains, including education, employment, working conditions, housing, income, education, and discrimination. They also had lower levels of: (1) community participation and social support; and (2) life satisfaction across all domains and lower levels of overall wellbeing, measured using SF-12 summary scores and the PWI-A.

Nearly seven percent of our sample was classified as disabled, however, there is a lack of international and national consensus on how best to measure disability in large-scale surveys [31–33]. As a result, disability prevalence estimates are strongly influenced by variation in conceptual or legal definitions of disability and the methods used to operationalise them. The majority of previous Australian surveys of disability, socio-economic conditions and health have relied on responses to a single question about disability which asked participants whether they had an impairment, long-term health condition, or disability which restricted everyday activities and lasted, or was likely to last, for a period of six months or more. Using this definition, the estimated prevalence of disability among 15 to 54 year old men was 18.1 % in the most recent wave of the Household, Income and Labour Dynamics in Australia Survey (HILDA) (unpublished analysis). The ABS Survey of Disability, Ageing and Carers, which uses an extensive range of questions to determine disability status and characteristics, found the crude prevalence of any disability among 15 to 54 year old males was 11.2 % and the prevalence of severe disabilities was 2.3 % [20].

Our findings correspond with those of previous Australian studies using HILDA and the ABS surveys – the Survey of Disability, Ageing and Carers and General Household Social Survey [12, 22, 34–37]. However, this paper adds to the existing literature by covering a broader range of social and economic domains and indicators of health and wellbeing and using an internationally validated instrument to measure disability. Moreover, whereas previous studies have focused largely on health status measures, ours includes measures of subjective wellbeing or life satisfaction. While our estimates of the associations between disability and socio-economic disadvantage and disability and mental health are consistent with those found elsewhere, the differences we report are higher than reported previously. For example, previous analyses of HILDA have reported a 2–3 point lower MCS score among people with disabilities [35, 36], while we estimated a nine point difference (nearly one standard deviation) in Ten to Men. This difference probably reflects the way disability was coded using the Washington Group questions, where we only categorised people as disabled who reported ‘a lot of difficulty or could not do at all’ across at least one of the six core domains. Although HILDA uses SF-36 and Ten to Men uses SF-12, this is not a convincing explanation for the discrepancy because validation studies in European countries found very high correlations between the component scores derived from the SF-12 and SF-36 [38].

### Strengths and limitations

The strengths of the study are its national scope, large sample size, comprehensive range of measures across multiple domains of the social determinants of health, and use of well-validated measures of disability and wellbeing. This study used the Washington Group questions to classify disability, a measurement tool designed to improve disability statistics and enable better comparisons of data, which has been validated internationally. The Washington Group questions are a significant advance on this approach. Additionally, this study uses the PWI-A to measure wellbeing. No study has previously examined the relationship between disability and a well-validated measure of personal wellbeing. Wellbeing is a positive construct, which may have a different relationship with disability compared to outcomes describing poor health.

The study also has a number of limitations. The response fraction was 35 % and thus selection bias due to non-response was likely. Adult participants in Ten to Men were slightly older than the general population they represent, more likely to have been born in Australia and to live in regional areas (reflecting the oversampling of these areas). The question of discrimination did not ask about types of discrimination (e.g. race/ethnicity, gender, disability) and so we were unable to determine the extent to which people with disabilities were exposed to disability-related discrimination. We used the MCS and PCS of SF-12 to measure health status, however the SF-12 includes questions (e.g. difficulty climbing stairs) that overlap with Washington Group questions (e.g. difficulty walking) and therefore we would expect lower scores among people with disabilities. In contrast, the PWI-A is a global measure of satisfaction across a range of life domains and we demonstrate approximately one standard deviation difference in PWI-A between people with and without disabilities and substantial differences across all domains of life.

Because the adult Ten to Men questionnaire was administered as a self-complete postal questionnaire in English it is likely to under-represent men who have difficulty reading and responding to a questionnaire due to visual, intellectual or cognitive impairments, or for whom English is not their first language. This is likely to result in an underestimate of disability prevalence and reduce the generalisability of the results to the Australian population. The lower prevalence of disability among men not born in Australia and men who did not speak English at home may reflect the fact that people who migrate tend to be healthier.

Disability is a contested concept and approaches to measurement have varied substantially [32, 39]. Our definition of disability was based on the Washington Group questions. Caution must be exercised in interpreting comparisons of our prevalence estimates with those from prior Australian studies. We were unable to use ABS modules for disability in Ten to Men because they are designed to be administered face-to-face and the ABS Survey of Disability, Ageing and Carers is wider in scope and includes people living in private dwellings, self-care retirement villages and establishments providing long-term cared accommodation. A population-based Norwegian study of adults aged 20 to 67 years compared ten approaches to measurement of disability including modified versions of the Washington Group questions and the ABS short form module. They found low concordance between the Washington Group questions and the ABS short module (kappa 0.14); only 47 % of people defined as having a disability using either approach were classified as disabled using both (this was 63 % for severe disability) [40]. Because the Washington Group questions have four response categories describing severity of the disability, multiple disability definitions can be created with different prevalence estimates. The Washington Group recommended the use of a cut-off of ‘a lot of difficulty’ or ‘cannot do at all’ recorded for at least one of the core domains for use in reporting of disability statistics [32]. Other criticisms of the Washington Group questions include that that they are relatively insensitive to detection of people with mental health problems because emotional functioning is not included in the core domains, and they classify many people with mild to moderate disability as non-disabled, even though they experience important limitations in their daily lives [5, 33, 41]. As people with mental health problems are more likely to experience disadvantage and have poorer overall health than people without disabilities, we believe that our estimates of associations between disability and socio-economic disadvantage and health and wellbeing are conservative.

We could not examine whether the socio-economic disadvantage experienced by men with disabilities contributed to inequalities in health and wellbeing between men with and without disabilities because this analysis is cross-sectional, and socio-economic disadvantage is likely to be both a cause and consequence of disability. However, given the well-established knowledge base about the social determinants of health and wellbeing it is likely that the disadvantaged socio-economic circumstances of men with disabilities make a substantial contribution to their poorer health.

### Future research directions

We classified men as having a disability if they reported a lot of difficulty or could not do activities across one or more of six core domains. Future analyses could explore a range of different cut-offs and compare outcomes among men with different types of impairments as previous research has shown that people with psychological and intellectual impairments tend to fare worse on socio-economic and health outcomes than people with sensory and speech and physical impairments [12, 22].

Analysis of the wealth of data on health behaviours; mental and physical health and wellbeing; and health service use collected in Ten to Men will provide further insight into the extent of disability-related health inequalities in Australian men. Ten to Men presents a unique opportunity to explore the health and wellbeing of disabled males over the short and longer term. This is particularly important because Australia is undergoing massive reform in the delivery of disability services with the introduction of the National Disability Insurance Scheme (NDIS). The NDIS provides individualised funding and support to Australians with severe, permanent disabilities. Ten to Men will provide an opportunity to track whether the implementation of the NDIS reduces disability-related socio-economic and health inequalities. Finally, the Washington Group questions were included in the second wave of Ten to Men and thus it will be possible to identify the men who acquire a disability and assess the causes and consequences of disability acquisition.

### Policy directions

Our findings demonstrate that Australian men with disabilities might be considered a ‘vulnerable population’ because they experience concentrated disadvantage that places them at higher risk of poor health and wellbeing than their non-disabled peers [42, 43]. While the NDIS is a welcome policy reform it will not necessarily solve the social and economic disadvantage and poorer health and wellbeing of men with disabilities because it does not fund housing, employment services, education or health services which will continue to be funded and provided under current state and federal arrangements. This means that policies and services that can address the socio-economic disadvantage and poorer health and wellbeing of men with disabilities will happen outside the NDIS. The results of these analyses highlight the importance of addressing social and economic disadvantage to improve the health of men with disabilities. This will require cross-sectoral engagement beyond the disability and health sectors and responses tailored to the particular concerns of Australian men with disabilities.

## Conclusion

Adult men with disabilities experience considerable social and economic disadvantage and poorer health and wellbeing than their non-disabled peers. They have been an under-recognised group in public health research and policy and require specific attention. Ten to Men is a unique international resource that can be used to better understand the lives of Australian men with disabilities.

## Additional files

Additional file 1: Table S1. Missing observations for variables in the analysis (analytic sample, *n*=13,569). (DOCX 23 kb)
Additional file 2: Table S2. Population weighted age-adjusted linear regression coefficients comparing health outcomes for men with disability compared to those with no disability. (DOCX 17 kb)

## Abbreviations

ABS: Australian Bureau of Statistics
ATSI: Aboriginal and Torres Strait Islander
HILDA: Household Income & Labour Dynamics in Australia (panel study)
IRSD: Index of Relative Socio-economic Disadvantage
MCS: Mental Component Summary score of the Short Form 12
NDIS: National Disability Insurance Scheme
PCS: Physical Component Summary score of the Short Form 12
PWI-A: Personal Wellbeing Index—Adult
SA1: Statistical Area Level 1
SF-12: Short Form 12
