# Supplementary material for: Inequalities in socio-economic characteristics and health and wellbeing of men with and without disabilities: a cross-sectional analysis of the baseline wave of the Australian Longitudinal Study on Male Health
Source: BMC Public Health. 2016 Oct 31;16(Suppl 3):23–31. doi: 10.1186/s12889-016-3700-y (PMC5103237; doi:10.1186/s12889-016-3700-y)
Supplement: Additional file 1: Table S1. — Missing observations for variables in the analysis (analytic sample, n=13,569). (DOCX 23 kb) [file 12889_2016_3700_MOESM1_ESM.docx]

Table S1. Missing observations for variables in the analysis (analytic sample, n=13,569)

|  | Not missing | |  | Missing | |
| --- | --- | --- | --- | --- | --- |
|  | n | % |  | n | % |
| **Demographic variables** |  |  |  |  |  |
| Country of birth | 13569 | 100.0 |  | 0 | 0.0 |
| Language spoken at home | 13410 | 98.8 |  | 159 | 1.2 |
| Indigenous status | 13509 | 99.6 |  | 60 | 0.4 |
| Relationship status | 13465 | 99.2 |  | 104 | 0.8 |
| Area of residence | 13559 | 99.9 |  | 10 | 0.1 |
| **Socio-economic variables** |  |  |  |  |  |
| Education | 13182 | 97.1 |  | 387 | 2.9 |
| Household income | 11609 | 85.6 |  | 1960 | 14.4 |
| Labour force status | 13305 | 98.1 |  | 264 | 1.9 |
| Skill level | 10833 | 95.1 |  | 553 | 4.9 |
| Employment arrangements* | 11196 | 98.3 |  | 190 | 1.7 |
| Number of hours worked* | 10507 | 92.3 |  | 879 | 7.7 |
| Hours of work preferred* | 11189 | 98.3 |  | 197 | 1.7 |
| Access to paid leave* | 9559 | 84.0 |  | 1827 | 16.0 |
| Shortage of money | 13185 | 97.2 |  | 384 | 2.8 |
| Housing tenure | 13244 | 97.6 |  | 325 | 2.4 |
| Housing affordability | 11530 | 85.0 |  | 2039 | 15.0 |
| SEIFA | 13564 | 100.0 |  | 5 | 0.0 |
| **Social support & participation** |  |  |  |  |  |
| Social support | 13123 | 96.7 |  | 446 | 3.3 |
| Group membership | 13141 | 96.8 |  | 428 | 3.2 |
| Community service | 13146 | 96.9 |  | 423 | 3.1 |
| Community events | 13182 | 97.1 |  | 387 | 2.9 |
| Discrimination | 13043 | 96.1 |  | 526 | 3.9 |
| **Health and wellbeing** |  |  |  |  |  |
| SF-12 Physical Component Score | 12880 | 94.9 |  | 689 | 5.1 |
| SF-12 Mental Component Score | 12880 | 94.9 |  | 689 | 5.1 |
| Personal Wellbeing Index | 13043 | 96.1 |  | 526 | 3.9 |
| Standard of living | 13322 | 98.2 |  | 247 | 1.8 |
| Health | 13323 | 98.2 |  | 246 | 1.8 |
| Life achievements | 13305 | 98.1 |  | 264 | 1.9 |
| Personal relationships | 13316 | 98.1 |  | 253 | 1.9 |
| Safety | 13317 | 98.1 |  | 252 | 1.9 |
| Feeling part of a community | 13305 | 98.1 |  | 264 | 1.9 |
| Future security | 13320 | 98.2 |  | 249 | 1.8 |

*among 11,386 employed participants
