# Supplementary material for: Inequalities in socio-economic characteristics and health and wellbeing of men with and without disabilities: a cross-sectional analysis of the baseline wave of the Australian Longitudinal Study on Male Health
Source: BMC Public Health. 2016 Oct 31;16(Suppl 3):23–31. doi: 10.1186/s12889-016-3700-y (PMC5103237; doi:10.1186/s12889-016-3700-y)
Supplement: Additional file 2: Table S2. — Population weighted age-adjusted linear regression coefficients comparing health outcomes for men with disability compared to those with no disability. (DOCX 17 kb) [file 12889_2016_3700_MOESM2_ESM.docx]

Table S2. Population weighted age-adjusted linear regression coefficients comparing health outcomes for men with disability compared to those with no disability

|  | Coeff | 95% CI |
| --- | --- | --- |
| SF-12 Physical Component Score | -9.4 | -10.5, -8.4 |
| SF-12 Mental Component Score | -8.8 | -9.8, -7.7 |
| Personal Wellbeing Index | -16.8 | -18.7, -14.9 |
| Satisfaction with life domains |  |  |
| Standard of living | -1.4 | -1.7, -1.2 |
| Health | -2.3 | -2.5, -2.1 |
| Life achievements | -1.9 | -2.2, -1.7 |
| Personal relationships | -1.3 | -1.6, -1.0 |
| Safety | -1.4 | -1.7, -1.2 |
| Feeling part of a community | -1.8 | -2.0, -1.5 |
| Future security | -2.0 | -2.2, -1.7 |
